# Métastases cutanées vues au laboratoire d'anatomie pathologique à Lomé, Togo entre 2005 et 2014

**DOI:** 10.11604/pamj.2015.22.388.7223

**Published:** 2015-12-30

**Authors:** Tchin Darre, Bayaki Saka, Dadja Essoya Landoh, Abas Mouhari-Toure, Koffi Amegbor, Palokinam Pitché, Gado Napo-Koura

**Affiliations:** 1 Laboratoire d'Anatomie et Cytologie Pathologiques, CHU Sylvanus Olympio, Lomé, Togo; 2 Service de Dermatologie et IST, CHU Sylvanus Olympio, Lomé, Togo; 3 Organisation Mondiale de la Santé, Lomé, Togo

**Keywords:** Métastases cutanées, anatomopathologie, épidémiologie, Togo, Skin metastases, pathology, epidemiology, Togo

## Abstract

**Introduction:**

L'objectif de cette étude était de documenter le profil épidémiologique et histologique des métastases cutanées vues au laboratoire d'anatomie pathologique (LAP) de Lomé.

**Méthodes:**

Il s'agissait d'une étude descriptive et transversale portant sur des cas de métastases cutanées observées au LAP du CHU Sylvanus Olympio entre 2005 et 2014.

**Résultats:**

Au cours de cette période d’étude, nous avons recensé 32 cas de métastases cutanées représentant 3,2% (1005 cas) de l'ensemble des cas de métastases enregistrées au LAP. L’âge moyen des patients atteints de ces métastases cutanées était de 42,6 ans et le sex-ratio (F/H) de 2,2. Sur le plan macroscopique, ces métastases étaient nodulaires dans 15 cas, bourgeonnantes dans 12 cas, ulcéré dans 3 cas et ulcéro-bourgeonnant dans 2 cas. Les types histologiques étaient représentés par les adénocarcinomes (19 cas, 59,4%), les carcinomes épidermoïdes (8 cas, 25%), la maladie de Paget (3 cas, 9,4%), le carcinome à petites cellules du type neuroendocrine (un cas, 3,1%) et le mélanome (un cas, 3,1%). Selon le degré de différenciation, les métastases cutanées étaient bien différenciées dans 14 cas (56%). Les principales localisations de ces métastases cutanées étaient le thorax (11 cas, 34,4%) suivi de l'abdomen (10 cas, 31,3%). Les cancers primitifs étaient surtout d'origine mammaire (14 cas, 43,7%).

**Conclusion:**

Cette étude montre que les métastases cutanées sont relativement rares et les tumeurs malignes d'origine mammaire sont les tumeurs primitives prédominantes. Avec un pourcentage élevé des formes peu différenciées et celles indifférenciées, l'amélioration du plateau technique du LAP (immunohistochimie) permettra d'accroitre ses capacités diagnostiques.

## Introduction

Les métastases sont des localisations secondaires des tumeurs malignes et peuvent être révélatrices de la tumeur primitive [1]. Toutefois, du fait de leur rareté, très peu d’études ont été décrites par certains auteurs en occident [2, 3]. A notre connaissance, aucune étude n'a été menée au Togo sur ces métastases cutanées. Nous avons alors jugé nécessaire de mener cette étude afin de documenter le profil épidémiologique et histologique de ces métastases cutanées diagnostiquées au Laboratoire d'Anatomie Pathologique du CHU de Lomé.

## Méthodes

Il s'agissait d'une étude transversale et descriptive sur tous les dossiers (registres et comptes rendus d'examens) portant sur les métastases cutanées histologiquement diagnostiquées au LAP du CHU de Lomé de janvier 2005 à décembre 2014 soit une période de 10 ans. Au cours de cette période, tous les cas d'examen portant sur un prélèvement de peau (biopsie, exérèse, pièces opératoires) ont été colligés à partir des données des registres dudit laboratoire. Les résultats et comptes rendus d'examen de tous les cas colligés avaient fait l'objet d'une collecte à l'aide d'une fiche préétablie et validé par l’équipe de recherche. Les techniques d'examen étaient constituées de coupes incluses en paraffine (56°-60° C), puis colorées à l'hématéine éosine. Les variables étudiées étaient les données épidémiologiques (sexe, âge, siège de la métastase, provenance du malade) et les données histologiques (type histologique).

### Considération éthique

Cette étude a reçu l'autorisation du Chef du département des laboratoires pour être conduite. Vu qu'il s'agissait de dépouillement des dossiers, le consentement des patients n’était pas requis. Toutefois lors du dépouillement et la collecte de données les noms des patients avaient été codés sur les fiches de collecte afin de préserver la confidentialité.

## Résultats

### Aspects épidémiologiques

Au cours de la période d’étude, nous avons recensé 32 cas de métastases cutanées représentant 3,2% (1005 cas) de l'ensemble des cas de métastases répertoriées, représentant une fréquence annuelle de 3,2 cas. L’âge moyen des patients atteints de ces métastases cutanées était de 42,6 ans, avec des extrêmes allant de 17 à 89 ans et le sex-ratio (F/H) de 2,2. Les patients de la tranche d’âge de 40 à 50 ans étaient les plus représentés (21 cas, 65,6%). Chez l'homme, la moyenne d’âge était de 40,1 ans et chez la femme, de 47,4ans. Les prélèvements étaient constitués de 17 biopsies cutanées et de 13 pièces opératoires. Ces prélèvements provenaient des services de dermatologie (16 cas), chirurgie (14 cas) et de gynécologie (2 cas).

### Aspects anatomopathologiques des métastases

Sur le plan macroscopique, ces métastases étaient nodulaires dans 15 cas, bourgeonnantes dans 12 cas, ulcérées dans 3 cas et ulcéro-bourgeonnantes dans deux cas. Les types histologiques étaient représentés par l'adénocarcinome (19 cas, 59,4%), le carcinome épidermoïde (8 cas, 25%), la maladie de Paget (3 cas, 9,4%), le carcinome à petites cellules du type neuroendocrine (un cas, 3,1%), et le mélanome (un cas, 3,1%). Selon le degré de différenciation, ces métastases étaient bien différenciées dans 14 cas, peu différenciées dans 9 cas, moyennement différenciées dans 5 cas et indifférenciées dans 4 cas.

### Localisation des métastases

Les localisations de ces métastases cutanées étaient le thorax principalement à sa face antérieure (11 cas, 34,4%), l'abdomen (10 cas, 31,3%), la tête et le cou (6 cas, 17,6%) et les membres (5 cas, 15,7%). Chez la femme, le thorax occupait le premier rang avec 9 cas suivi de l'abdomen 4 cas alors que chez l'homme, l'abdomen était en première position avec 6 cas (Tableau 1). Les cancers primitifs étaient essentiellement d'origine mammaire (14 cas, 43,7%) et digestive (8 cas, 25%) dans les deux sexes (Tableau 2).

**Tableau 1:** Répartition des métastases cutanées selon la topographie vues au laboratoire d'anatomie pathologique de Lomé entre 2005 et 2014

	Femme	Homme	Effectif	Pourcentage (%)
**Thorax**	9	2	11	34,4
**Abdomen**	4	6	10	31,3
**Tête et cou**	5	1	6	17,6
**Membres inférieurs**	2	1	3	9,4
**Membres supérieurs**	2	0	2	6,3
**Total**	22	10	32	100

**Tableau 2:** Répartition des métastases cutanées en fonction du siège de la tumeur primitive, laboratoire d'anatomie pathologique de Lomé entre 2005 et 2014

	Femme	Homme	Effectif	Pourcentage (%)
**Sein**	1 3	1	14	43,7
**Tube digestif**	3	5	8	25,0
**Utérus**	3	0	3	9,3
**Thyroïde**	1	1	2	6,3
**Prostate**	0	2	2	6,3
**Broncho-pulmonaire**	1	1	2	6,3
**Cavité buccale**	1	0	1	3,1
**Total**	22	10	32	100

## Discussion

### Aspects épidémiologiques

Au cours de la période d’étude, 32 cas des métastases cutanées sont diagnostiquées au LAP du CHU de Lomé, ce qui nous permet de conclure que la fréquence d'apparition des métastases cutanées est relativement faible. Ce résultat rejoint certaines données de la littérature sur lesquels le foie est concerné 10 fois plus souvent que la peau [4]. Cependant, pour certains auteurs la proportion des métastases cutanées est vraisemblablement plus grande qu'il n'est classique de la dire et s’échelonne entre 2 et 10% des métastases [5]. Ainsi, Alcaraz *et al*. avaient rapporté dans leur série une fréquence des métastases cutanées de 2% de l'ensemble des métastases [6]. L’âge de la population la plus touchée par les métastases cutanées est celui compris entre 40 et 50 ans. Nos résultats rejoignent ceux qui ont établi que l'existence des métastases cutanées était plus élevée entre la quatrième et la sixième décennie de la vie [7]. Ainsi pour ces auteurs, la fréquence suivant l’âge paraît en relation avec celle des cancers eux-mêmes. Cependant, certaines tumeurs primitives de l'enfant ou du sujet jeune peuvent avoir une expression cutanée [8]. Nous avons noté une prédominance féminine, ce qui s'explique par le fait que dans notre étude l’étiologie la plus fréquente est le cancer primitif du sein qui est l'apanage des femmes. Nos résultats sont conforment aux travaux de Moore [9] et de Georgiannos *et al*. [10] qui ont montré une incidence deux fois plus grande des métastases cutanées chez la femme que chez l'homme.

### Aspects anatomopathologiques

Sur le plan macroscopique, l'aspect nodulaire était le plus représenté concordant avec les données de la littérature [2, 11]. Néanmoins, nous pensons que dans notre étude, il s'agissait au préalable de nodules qui auraient évolués vers les formes bourgeonnantes, lié au fait que la majorité des patients consultent le plus souvent tardivement. Le type histologique le plus retrouvé était l'adénocarcinome (59,4%) suivi du carcinome épidermoïde (25%). Il est difficile de tirer des conclusions sur cette donnée. En effet, les adénocarcinomes représentent le type histologique le plus fréquent au niveau du sein et du tube digestif, siège de la majorité des tumeurs primitives dont nous étudions les localisations secondaires cutanées. Nos résultats concordent néanmoins avec les données des travaux de Bansal *et al*. qui avaient rapporté que la tumeur primitive était un adénocarcinome dans trois-quarts des cas et un carcinome épidermoïde dans un quart des cas [12]. Nous n'avons recensé que trois cas de maladie de Paget du mamelon secondaire à un adénocarcinome mammaire, confirmant ainsi la rareté de ce type histologique, déjà signalé dans la littérature [13]. De même, le mélanome a été rare dans notre série confirmant la faible fréquence de ce cancer chez les sujets à peau pigmentée [14]. Selon le degré de différenciation, 14 cas étaient bien différenciés contre neuf cas peu différenciés et quatre cas indifférenciés. Le degré de différenciation de la métastase peut permettre de retrouver la tumeur primitive. Ainsi, dans le cas où la métastase est moyennement ou bien différenciée par rapport à la tumeur primitive, on la rattache au cancer primitif déjà diagnostiqué. Par contre, lorsque la métastase est peu différenciée ou indifférenciée, l'immunohistochimie s'avère nécessaire pour identifier l'origine cellulaire de la tumeur primitive [6]. La majorité des tumeurs primitives siégeaient au niveau du sein (43,7%) et du tube digestif (25%). Chez la femme, le sein est l'organe le plus concerné (13 cas) et chez l'homme le tube digestif (6 cas). Selon Moore et al. [9], le sein représente le site d'origine quasi constant chez la femme tandis que chez l'homme des carcinomes bronchiques sont le plus souvent en cause, suivi par les mélanomes. Toujours selon ses auteurs, la fréquence des tumeurs primitives en cause est en rapport avec le sexe et avec celle des cancers eux-mêmes. Cependant, aucune tumeur primitive n'a de tendance sélective à disséminer au niveau de la peau. Ainsi, des cas isolés de métastases cutanées provenant de toute localisation tumorale primitive sont rapportés [10, 15]. Les sièges de prédilection des métastases cutanées dans notre étude étaient la face antérieure du thorax (34,4%) et l'abdomen (31,3%). Selon Röösli *et al*. [16] et Nggada *et al*. [17], trois fois sur quatre, les lésions cutanées secondaires siègent plus sur la face antérieure du thorax et l'abdomen chez la femme et sur la tête et le cou chez l'homme.

## Conclusion

Notre étude nous a permis de noter une fréquence relativement faible des métastases cutanées au CHU de Lomé avec une prédominance féminine et fréquence élevée des formes peu ou indifférenciées. Ces données nous permettent de plaider pour l'amélioration du plateau technique du LAP (immunohistochimie) et la mise en place d'un registre de cancer.
